# Amplified inflammatory and immune responses in viral-associated pulmonary aspergillosis

**DOI:** 10.3389/fcimb.2026.1850127

**Published:** 2026-05-28

**Authors:** Xue Li, Guiying Xu, Jiancun Cao, Ruyu Yang, Shasha Fu, Zhonghua Qin, Lijia Xie, Hongxia Shao, Junping Wu, Huaiyong Chen

**Affiliations:** 1 Tianjin Key Laboratory of Lung Regenerative Medicine, Haihe Hospital, Tianjin University, Tianjin, China; 2 Department of Tuberculosis, Haihe Clinical College, Tianjin Medical University, Tianjin, China; 3 Department of Critical Care Medicine, Haihe Clinical College, Tianjin Medical University, Tianjin, China; 4 Center for Accurate Detection of Tuberculosis, Haihe Hospital, Tianjin University, Tianjin, China; 5 Key Research Laboratory for Infectious Disease Prevention for State Administration of Traditional Chinese Medicine, Tianjin Institute of Respiratory Diseases, Tianjin, China

**Keywords:** aspergillus infection, immune dysregulation, metabolic reprogramming, oxidative stress, viral pneumonia

## Abstract

**Background:**

Viral-associated pulmonary aspergillosis (VAPA) is a severe complication of viral pneumonia (VP) that is associated with pronounced inflammatory amplification, immune dysregulation, and increased mortality. However, systemic metabolic and immune response patterns accompanying VAPA remain incompletely understood.

**Methods:**

Plasma samples were obtained from 35 patients with viral pneumonia (VP group) and 20 with viral-associated pulmonary aspergillosis (VAPA group). An integrated multi-omics strategy combining data-independent acquisition (DIA)–based proteomics and untargeted metabolomics was used. In total, 1,930 proteins and 1,532 metabolites were identified. Differential analyses along with Gene Ontology (GO) and Kyoto Encyclopedia of Genes and Genomes (KEGG) pathway enrichment analyses were conducted to characterize host immune and metabolic alterations associated with viral–fungal coinfection.

**Results:**

Compared to patients with VP, those with VAPA showed more pronounced systemic inflammatory activity, immune dysregulation, hepatic and renal impairment, and coagulation abnormalities. Proteomic profiling revealed a higher abundance of proteins related to antioxidant responses, protein degradation pathways, and inflammatory and immune signaling in patients with VAPA. Metabolomic analyses indicated substantial alterations in lipid metabolism, increased oxidative stress–related metabolites, and dysregulation of hormone- and vitamin-associated metabolic pathways. Together, these proteomic and metabolomic patterns were associated with enhanced inflammatory burden, disrupted immune regulation, and greater disease severity.

**Conclusions:**

This study provides a systematic overview of immune and metabolic alterations in patients with VAPA. The observed multi-omics features offer insights into host responses associated with viral–fungal coinfection and provide potential theoretical support for early identification and targeted intervention of VAPA.

## 1 Introduction

Respiratory viral infections remain a major global public health challenge and can lead to varying degrees of lower respiratory tract injury, acute lung injury, and even multi-organ dysfunction (Su et al., 2024; Zheng et al., 2025). Viral pneumonia (VP) has been recognized as one of the leading causes of severe respiratory infections and is associated with high mortality worldwide (Flerlage et al., 2021; Hanage and Schaffner, 2025; Iuliano et al., 2018). In recent years, advances in intensive care management and pathogen detection techniques have revealed that a subset of patients with viral pneumonia may develop secondary fungal infections, particularly invasive pulmonary aspergillosis (IPA) (Beltrame et al., 2023; Gioia et al., 2024; Lu et al., 2024). Viral-associated pulmonary aspergillosis (VAPA), which mainly encompasses influenza-associated pulmonary aspergillosis (IAPA) and COVID-19-associated pulmonary aspergillosis (CAPA), has emerged as a clinically important complication of severe respiratory viral infection and is associated with reported mortality rates exceeding 50%, substantially higher than those observed in patients with VP alone (Beltrame et al., 2023; Feys et al., 2024a). Previous studies have shown that VAPA is closely associated with exaggerated inflammatory responses, extensive lung tissue injury, and adverse clinical outcomes (Feys et al., 2024a). Current research on VAPA has primarily focused on epidemiological characteristics, radiological features, and antifungal treatment strategies; however, molecular mechanisms underlying the markedly aggravated host injury following viral–*Aspergillus* coinfection remain incompletely understood.

Respiratory viral infections can disrupt host immune homeostasis through multiple mechanisms. It is well documented that influenza viruses and SARS-CoV-2 can impair integrity of the respiratory epithelial barrier while simultaneously compromising phagocytic and microbicidal functions of neutrophils, monocytes, and alveolar macrophages, thereby increasing host susceptibility to *Aspergillus* invasion (Arastehfar et al., 2020; Feldman and Anderson, 2021; Feys et al., 2022, 2024b; Liu et al., 2022; Salazar et al., 2022; Tavakoli et al., 2020). *Aspergillus* species can further activate host immune cells via β-glucans and secreted proteases, inducing robust production of pro-inflammatory mediators such as TNF-α, IL-6, IL-8, MIP-2, and MCP-1, which ultimately drives inflammatory amplification and immune dysregulation (Hohl et al., 2005; Pan and Lee, 2024; Tomee et al., 1997; Zoran et al., 2022). Collectively, viral–fungal coinfection may promote rapid disease progression through a synergistic process involving epithelial barrier disruption, impaired immune defense, and fungus-driven inflammation. With the development of systems biology approaches, host metabolic reprogramming has been increasingly recognized as a central mechanism that regulates immune responses during viral and fungal infections. Viral infections have been reported to induce enhanced glycolysis, lipid metabolic disturbances, and redox imbalance, thereby influencing immune cell energy supply, migratory capacity, cytokine production, and efficiency of pathogen clearance (Chandler et al., 2016; Cui et al., 2016; Li et al., 2022, 2023, 2025; Shi et al., 2024; Zhao et al., 2022). Similarly, fungal infection is accompanied by substantial metabolic perturbations and oxidative stress, which may further amplify inflammatory responses and contribute to immune imbalance (Abdelmoneim et al., 2025; Tomee et al., 1997; Zoran et al., 2022). Despite these observations, systematic comparisons of proteomic and metabolomic alterations between patients with VP and those with VAPA remain limited, and the global landscape of metabolic–immune interactions associated with viral–fungal coinfection is not yet well defined.

In the present study, integrated data-independent acquisition (DIA)-based proteomic and untargeted metabolomic analysis was performed on plasma samples obtained from 35 patients with viral pneumonia (VP group) and 20 patients with viral-associated pulmonary aspergillosis (VAPA group). Through comprehensive multi-omics integration, this study aimed to delineate host immune response alterations and metabolic reprogramming associated with viral–fungal coinfection and to identify VAPA-specific proteomic and metabolic features. These findings may contribute to improved understanding of the molecular basis of VAPA and provide a framework for the exploration of potential biomarkers and therapeutic targets.

## 2 Materials and methods

### 2.1 Study design and participants

This retrospective observational study included patients diagnosed with viral pneumonia who were admitted to Tianjin Haihe Hospital in China between May 2023 and January 2025. VP was diagnosed according to the Diagnosis and Treatment Protocol for Influenza (2023 Edition) and the Diagnosis and Treatment Protocol for Coronavirus Disease 2019 (COVID-19) (Trial Version 10), both issued by the National Health Commission of China. Based on the presence or absence of Aspergillus infection, patients were classified into VP group (n = 35) and VAPA group (n = 20). The VP group consisted exclusively of patients with COVID-19-associated viral pneumonia. The VAPA group included 11 patients with IAPA and 9 patients with CAPA. IAPA was diagnosed according to the 2020 expert opinion case definition (Verweij et al., 2020), and CAPA was diagnosed according to the 2020 ECMM/ISHAM consensus criteria (Koehler et al., 2021). None of the patients in the VAPA group received antifungal prophylaxis prior to diagnostic sampling. All included patients were previously immunocompetent and had no classical immunocompromising host factors, including hematological malignancy, transplantation, HIV infection, prolonged neutropenia, or long-term use of systemic corticosteroids or immunosuppressive agents before admission.

### 2.2 Plasma sample collection

Peripheral venous blood samples were collected at hospital admission, when all included patients had already been diagnosed with viral pneumonia. Patients were subsequently classified into the VP or VAPA group according to the final diagnosis of *Aspergillus* infection during hospitalization. Blood samples were drawn into EDTA anticoagulant tubes and centrifuged at 3,000 rpm for 10 min at 4 °C to separate plasma. The resulting supernatants were aliquoted and stored at −80 °C until proteomic and metabolomic analyses.

### 2.3 Clinical data and laboratory parameters

Demographic information, including age and sex, as well as clinical manifestations, was collected from all enrolled patients. Laboratory parameters presented in **Table 1** were obtained at the same time point as plasma sampling, namely at hospital admission. These parameters complete blood counts, inflammatory markers, liver and renal function indices, and coagulation parameters. All clinical data were extracted from the electronic medical record system of the hospital and independently reviewed and verified by two investigators. All clinical data were extracted from the electronic medical record system of the hospital and independently reviewed and verified by two investigators.

**Table 1:** Demographic and clinical characteristics of patients with VP and VAPA.

Characteristics	VP (n=35)	VAPA (n=20)	*p* value
Sex			
Male	28 (80.0%)	16 (80.0%)	1.000
Female	7 (20.0%)	4 (20.0%)	1.000
Age, years	71.8 ± 8.1	72.4 ± 10.8	1.000
Symptoms			
Body temperature	37.3 ± 1.2	38.3 ± 1.0	0.027
Cough	33 (94.2%)	20 (100.0%)	0.276
Expectoration	26 (74.3%)	19 (95.0%)	0.055
Rhinorrhea	9 (25.7%)	3 (77.3%)	0.355
Dyspnea	10 (28.6%)	16 (80.0%)	< 0.001
Duration of hospitalization, days	11 (3, 25)	18 (4, 88)	0.001
28-day mortality	0 (0.0%)	9 (45.0%)	< 0.001

### 2.4 Plasma metabolomic analysis

UHPLC–MS/MS Ultra-high-performance liquid chromatography–tandem mass spectrometry (UHPLC–MS/MS) analysis was performed using the Vanquish UHPLC system coupled to an Orbitrap Q Exactive™ HF-X mass spectrometer (Thermo Fisher Scientific, Germany). All analyses were performed by Novogene Co., Ltd. (Beijing, China). Data were acquired in data-dependent MS/MS mode with polarity switching. As described previously (Li et al., 2023; Yan et al., 2022), pooled quality control (QC) samples were prepared by mixing equal aliquots of all plasma samples to equilibrate the LC–MS system, monitor instrument stability, and assess analytical reproducibility. QC samples were injected before, during, and after sample analysis. Moreover, blank samples were included to eliminate background signals. Metabolites with a coefficient of variation (CV) of > 30% in the QC samples were excluded. Peak extraction, alignment, and quantification were performed based on extracted ion chromatograms (XICs). Metabolite identification was performed using the Novogene in-house MS/MS spectral database (NovoMetDB) with a mass tolerance of 10 ppm. Peak areas were normalized using the following formula: normalized peak area = raw peak area/(total peak area of the sample/total peak area of QC1).

Metabolite annotation was performed using the KEGG, HMDB, and LIPID MAPS databases. Multivariate statistical analyses, including principal component analysis (PCA) and partial least squares discriminant analysis (PLS-DA), were conducted using the metaX package (Wen et al., 2017). Differential metabolites were identified based on the following criteria: variable importance in projection (VIP) > 1, *p* < 0.05, and fold change (FC) > 1.5. KEGG pathway enrichment analysis was conducted based on differential metabolites to explore disease-associated biological processes.

### 2.5 Plasma proteomic analysis

Total plasma proteins were extracted using SpinTip beads, and protein concentrations were determined using the bicinchoninic acid (BCA) assay. LC–MS/MS analysis was performed on a Vanquish Neo UHPLC system coupled with the Orbitrap Astral mass spectrometer (Thermo Fisher Scientific, Germany) operating in data-independent acquisition (DIA) mode. All proteomic analyses were performed by Novogene Co., Ltd. (Beijing, China). Raw DIA data were processed using the DIA-NN software. Protein annotation was performed using the InterProScan software. Differentially expressed proteins were identified based on the criteria of *p* < 0.05 and fold change (FC) > 1.5. KEGG and GO pathway enrichment analysis was conducted based on differential metabolites to explore disease-associated biological processes.

### 2.6 Statistical analysis

Normality of data distribution was assessed using the Shapiro–Wilk test. Continuous variables with a normal distribution are presented as the mean ± standard deviation (SD), whereas non-normally distributed variables are presented as the median with interquartile range (Q1, Q3). Categorical variables are expressed as frequencies and percentages (%). Comparisons between two groups were performed using Student’s *t*-test for normally distributed variables or the appropriate non-parametric test for non-normally distributed variables. A *p*-value < 0.05 was considered statistically significant. Data visualization, including volcano plots, heatmaps, and bubble plots, was performed using the ggplot2 package in R, while box plots were generated using GraphPad Prism 9.

## 3 Results

### 3.1 Demographic characteristics and clinical manifestations

Clinical data from 35 patients with VP and 20 patients with VAPA were retrospectively analyzed. Demographic characteristics and baseline clinical features are summarized in **Table 1**. No significant differences were observed between the two groups with respect to age or sex distribution. Comparative analysis of clinical manifestations indicated that patients in the VAPA group had a higher body temperature than those in the VP group (38.3 ± 1.0 vs. 37.3 ± 1.2 °C, *P* = 0.027). The incidence of dyspnea was also increased in the VAPA group than in the VP group (80.0% vs. 28.6%, *P* < 0.001). In addition, patients with VAPA had a longer duration of hospitalization (18 vs. 11 days, *P* = 0.001) and a higher 28-day mortality rate (45.0% vs. 0%, *P* < 0.001) than those with VP, indicating a more severe clinical course.

Laboratory findings are summarized in **Table 2**. Compared to those in the VP group, patients in the VAPA group exhibited higher white blood cell and neutrophil counts, along with increased levels of interleukin-6 (IL-6) and procalcitonin (PCT), consistent with a more pronounced systemic inflammatory response. Regarding liver function, alanine aminotransferase (ALT) and aspartate aminotransferase (AST) levels were higher in the VAPA group than in the VP group, whereas serum albumin and cholinesterase levels were lower, suggesting impaired hepatic synthetic capacity. Renal and metabolic indices showed elevated urea levels and reduced uric acid levels in patients with VAPA. In addition, coagulation test revealed prolonged thrombin time and increased D-dimer levels in the VAPA group, indicating abnormalities in the coagulation status. Taken together, these clinical and laboratory findings indicate that patients with VAPA are characterized by enhanced systemic inflammation, involvement of multiple organ systems, and coagulation disturbances compared to patients with VP alone.

**Table 2:** Laboratory parameters of patients with VP and VAPA.

Characteristics	VP (n=35)	VAPA (n=20)	*p* value
Hs-CRP (mg/L; NR 0-10)	14.27 (0.50, 194.00)	16.96 (0.5, 178.8)	0.727
IL-6 (ng/L; NR 0-10)	10.7 (1.5, 214.5)	26.0 (6.5, 221.5)	< 0.001
PCT (ng/mL; NR 0-0.5)	0.054 (0.04, 1.584)	0.144 (0.044, 1.740)	< 0.001
WBC (10^9/L; NR 4-10)	7.55 (3.25, 16.57)	9.7 (4.86, 32.83)	0.003
NEU (10^9/L; NR 1.8-6.3)	5.2 (2.09, 15.49)	7.88 (3.96, 29.11)	0.004
LYM (10^9/L; NR 1.1-3.2)	0.86 (0.17, 3.12)	0.74 (0.32, 2.81)	0.167
MON (10^9/L; NR 0.1-0.6)	0.51 (0.15, 1.02)	0.72 (0.12, 2.93)	0.058
EOS (10^9/L; NR 0.02-0.52)	0.00 (0.00, 0.17)	0.00 (0.00, 0.10)	0.160
BASO (10^9/L; NR 0-0.06)	0.01 (0.00, 0.05)	0.02 (0.00, 0.07)	0.008
RBC (10^12/L; NR 3.8-5.1)	4.34 ± 0.52	3.58 ± 0.81	0.006
Hgb (g/L; NR 115-150)	132.43 ± 16.58	109.00 ± 25.60	0.006
PLT (10^9/L; NR 125-350)	173 (46, 403)	196 (85, 597)	0.993
NRBC (10^9/L; NR)	0.00 (0.00, 0.01)	0.00 (0.00, 0.08)	0.021
IG (10^9/L; NR 0-0.06)	0.03 (0.01, 0.57)	0.08 (0.02, 1.49)	0.004
TP (g/L; NR 63-82)	65.05 ± 7.01	57.15 ± 7.64	0.004
ALB (g/L; NR 35-50)	33.89 ± 4,62	27.99 ± 5.00	< 0.001
ALT (U/L; NR 0-35)	26.5 (16, 70)	51 (15, 169)	0.042
AST (U/L; NR 14-36)	32 (17, 65)	46 (15, 210)	0.027
CHE (U/L; NR 4650-10440)	5559.50 ± 1492.61	3582.29 ± 1455.81	< 0.001
UA (μmol/L; NR 149-369)	251.86 ± 99.18	173.94 ± 78.23	0.022
UREA (mmol/L; NR 2.5-6.1)	5.98 ± 2.12	10.42 ± 4.72	0.001
FIB (g/L; NR 2-4)	4.59 (2.18, 9.09)	4.20 (2.10, 7.92)	0.970
TT (s; NR 16-26)	17.00 (14.8, 26.9)	24.1 (15.1, 57.5)	0.002
DD (ng/mL; NR 0-0.243)	0.46 (0.11, 18.908)	1.146 (0.078, 5.02)	0.014

Hs-CRP, High-sensitivity C-reactive protein; IL-6, Interleukin-6; PCT, Procalcitonin; WBC, White Blood Cell; NEU, Neutrophil Count; LYM, Lymphocyte Count; MON, Monocyte Count; EOS, Eosinophil Count; BASO, Basophil Count; RBC, Red Blood Cell; Hgb; Hemoglobin; PLT; Platelet; NRBC, Nucleated Red Blood Cell; IG, Immature Granulocytes; TP, Total Protein; ALB, Albumin; ALT, Alanine Aminotransferase; AST, Aspartate Aminotransferase; CHE, Cholinesterase; UA, Uric Acid; UREA, Urea; FIB, Fibrinogen; TT, Thrombin Time; DD, D-dimer.

To further assess whether the clinical differences observed in the overall VP versus VAPA comparison were influenced by viral etiology, we performed an additional comparison between COVID-19-associated VP patients and CAPA patients. Consistent with the main analysis, CAPA patients showed higher body temperature, a higher frequency of dyspnea, longer hospitalization, and higher 28-day mortality than VP patients (**Table 3**). Laboratory analysis also showed increased IL-6, PCT, neutrophil count, and immature granulocyte count, reduced lymphocyte count, lower albumin and cholinesterase levels, and prolonged thrombin time in the CAPA group (**Table 4**). These findings suggest that the more severe clinical and inflammatory phenotype observed in the VAPA group was also evident in the CAPA subgroup within the same COVID-19 viral background.

**Table 3:** Demographic and clinical characteristics of patients with VP and CAPA.

Characteristics	VP (n=35)	CAPA (n=9)	*p* value
Sex			
Male	28 (80.0%)	6 (66.7%)	0.685
Female	7 (20.0%)	3 (33.3%)	0.685
Age, years	71.8 ± 8.1	73.5 ± 11.8	0.793
Symptoms			
Body temperature	37.3 ± 1.2	38.3 ± 0.9	0.034
Cough	33 (94.2%)	9 (100.0%)	1.000
Expectoration	26 (74.3%)	9 (100.0%)	0.214
Rhinorrhea	9 (25.7%)	1 (11.1%)	0.627
Dyspnea	10 (28.6%)	9 (100.0%)	< 0.001
Duration of hospitalization, days	11 (3, 25)	14 (8, 70)	0.032
28-day mortality	0 (0.0%)	4 (44.4%)	< 0.001

**Table 4:** Laboratory parameters of patients with VP and CAPA.

Characteristics	VP (n=35)	CAPA (n=9)	*p* value
Hs-CRP (mg/L; NR 0-10)	15.17 (0.50, 194.00)	21.32 (0.5, 147.74)	0.938
IL-6 (ng/L; NR 0-10)	10.1 (1.5, 214.5)	25.0 (9.8, 50.3)	0.017
PCT (ng/mL; NR 0-0.5)	0.14 ± 0.28	0.42 ± 0.63	0.002
WBC (10^9/L; NR 4-10)	7.55 (3.25, 16.57)	9.48 (6.77, 13.70)	0.061
NEU (10^9/L; NR 1.8-6.3)	5.2 (2.09, 15.49)	8.27 (5.79, 12.43)	0.013
LYM (10^9/L; NR 1.1-3.2)	0.86 (0.17, 3.12)	0.59 (0.40, 0.92)	0.04
MON (10^9/L; NR 0.1-0.6)	0.51 (0.15, 1.02)	0.76 (0.23, 0.86)	0.300
EOS (10^9/L; NR 0.02-0.52)	0.16 ± 0.03	0.13 ± 0.04	0.856
BASO (10^9/L; NR 0-0.06)	0.14 ± 0.12	0.19 ± 0.02	0.656
RBC (10^12/L; NR 3.8-5.1)	4.32 (3.20, 5.78)	4.02 (3.45, 5.05)	0.027
Hgb (g/L; NR 115-150)	129 (90, 176)	120 (103, 148)	0.015
PLT (10^9/L; NR 125-350)	173 (46, 403)	225.5 (89, 395)	0.405
NRBC (10^9/L; NR)	0.00 (0.00, 0.01)	0.01 (0.00, 0.01)	0.006
IG (10^9/L; NR 0-0.06)	0.03 (0.01, 0.57)	0.07 (0.04, 0.84)	< 0.001
TP (g/L; NR 63-82)	64.2 (49.6, 82.0)	60.5 (52.7, 72.3)	0.081
ALB (g/L; NR 35-50)	35.1 (25.6, 41.0)	28.6 (24.0, 41.5)	0.012
ALT (U/L; NR 0-35)	26.0 (11, 91)	56 (15, 101)	0.148
AST (U/L; NR 14-36)	31.5 (8, 65)	30 (15, 104)	0.446
CHE (U/L; NR 4650-10440)	5820 (3024,7890)	3648(1960, 6381)	0.005
UA (μmol/L; NR 149-369)	230.5 (93, 443)	221(87, 329)	0.152
UREA (mmol/L; NR 2.5-6.1)	5.64 (1.95, 11.7)	9.73 (2.41, 18.55)	0.092
FIB (g/L; NR 2-4)	4.59 (2.18, 9.09)	4.20 (2.10, 7.45)	0.951
TT (s; NR 16-26)	18.5 ± 3.29	22.3 ± 5.08	0.017
DD (ng/mL; NR 0-0.243)	1.14 ± 3.13	1.07 ± 1.20	0.951

Hs-CRP, High-sensitivity C-reactive protein; IL-6, Interleukin-6; PCT, Procalcitonin; WBC, White Blood Cell; NEU, Neutrophil Count; LYM, Lymphocyte Count; MON, Monocyte Count; EOS, Eosinophil Count; BASO, Basophil Count; RBC, Red Blood Cell; Hgb, Hemoglobin; PLT, Platelet; NRBC, Nucleated Red Blood Cell; IG, Immature Granulocytes; TP, Total Protein; ALB, Albumin; ALT, Alanine Aminotransferase; AST, Aspartate Aminotransferase; CHE, Cholinesterase; UA, Uric Acid; UREA, Urea; FIB, Fibrinogen; TT, Thrombin Time; DD, D-dimer.

### 3.2 Global plasma proteomic and metabolomic profiles

**Figure 1:**
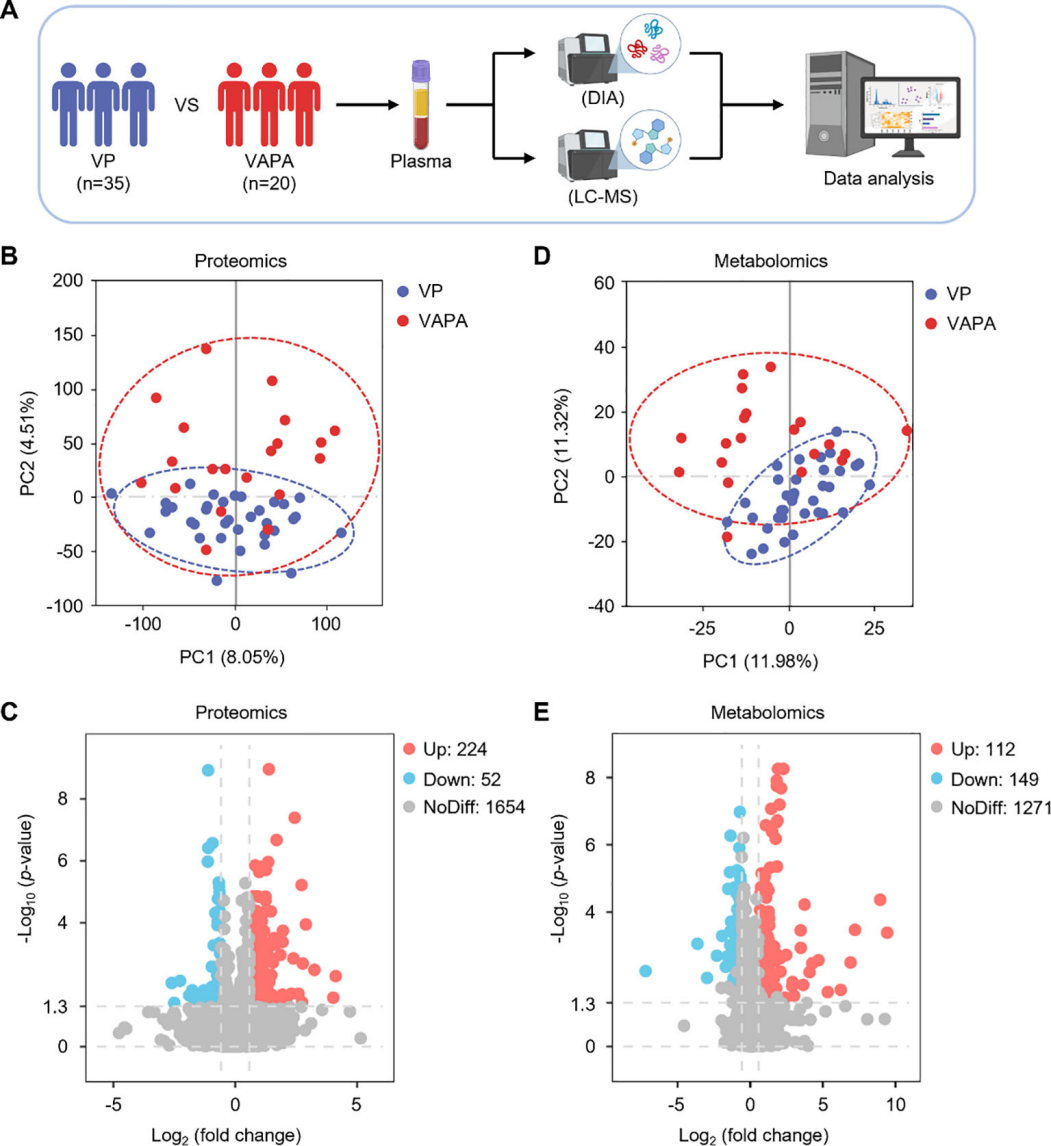
Study design and global multi-omics profiling of patients with VP and VAPA. **(A)** Schematic overview of the study design and patient grouping. **(B)** Principal component analysis (PCA) of plasma proteomic datasets. Each dot represents an individual plasma sample and different colors denote different patient groups. **(C)** Volcano plots of differentially expressed proteins between the VP and VAPA groups. Red points indicate significantly increased features, blue points indicate significantly decreased features, and gray points represent features with no significant differences. **(D)** Principal component analysis (PCA) of plasma metabolomic datasets. Each dot represents an individual plasma sample and different colors denote different patient groups. **(E)** Volcano plots of differentially expressed metabolites between the VP and VAPA groups. Red points indicate significantly increased features, blue points indicate significantly decreased features, and gray points represent features with no significant differences.

To characterize systemic metabolic and immune alterations associated with VAPA, DIA-based proteomic and untargeted metabolomic analyses were performed on plasma samples obtained from patients in both the VAPA and VP groups (**Figure 1A**). In total, 1,930 plasma proteins were quantified. PCA of the proteomic data also revealed differences between the VP and VAPA groups, suggesting dissimilarities in the overall protein expression patterns (**Figure 1B**). Differential protein analysis identified 276 proteins with significantly altered abundance, of which 224 were increased and 52 were decreased in the VAPA group relative to those in the VP group (**Figure 1C**). Metabolomic profiling was performed and 1,532 metabolites were identified through plasma metabolomic profiling. Principal component analysis (PCA) indicated differences between the VP and VAPA groups based on global metabolic profiles (**Figure 1D**). Differential metabolite analysis identified 261 metabolites with significantly altered abundance, of which 112 metabolites were increased and 149 metabolites were decreased in the VAPA group compared to those in the VP group (**Figure 1E**). Taken together, these findings indicate that patients with VAPA exhibit coordinated alterations in both protein expression profiles and plasma metabolite composition, reflecting broad changes in systemic metabolic and immune-related processes.

### 3.3 Plasma proteomic characteristics of patients with VAPA

**Figure 2:**
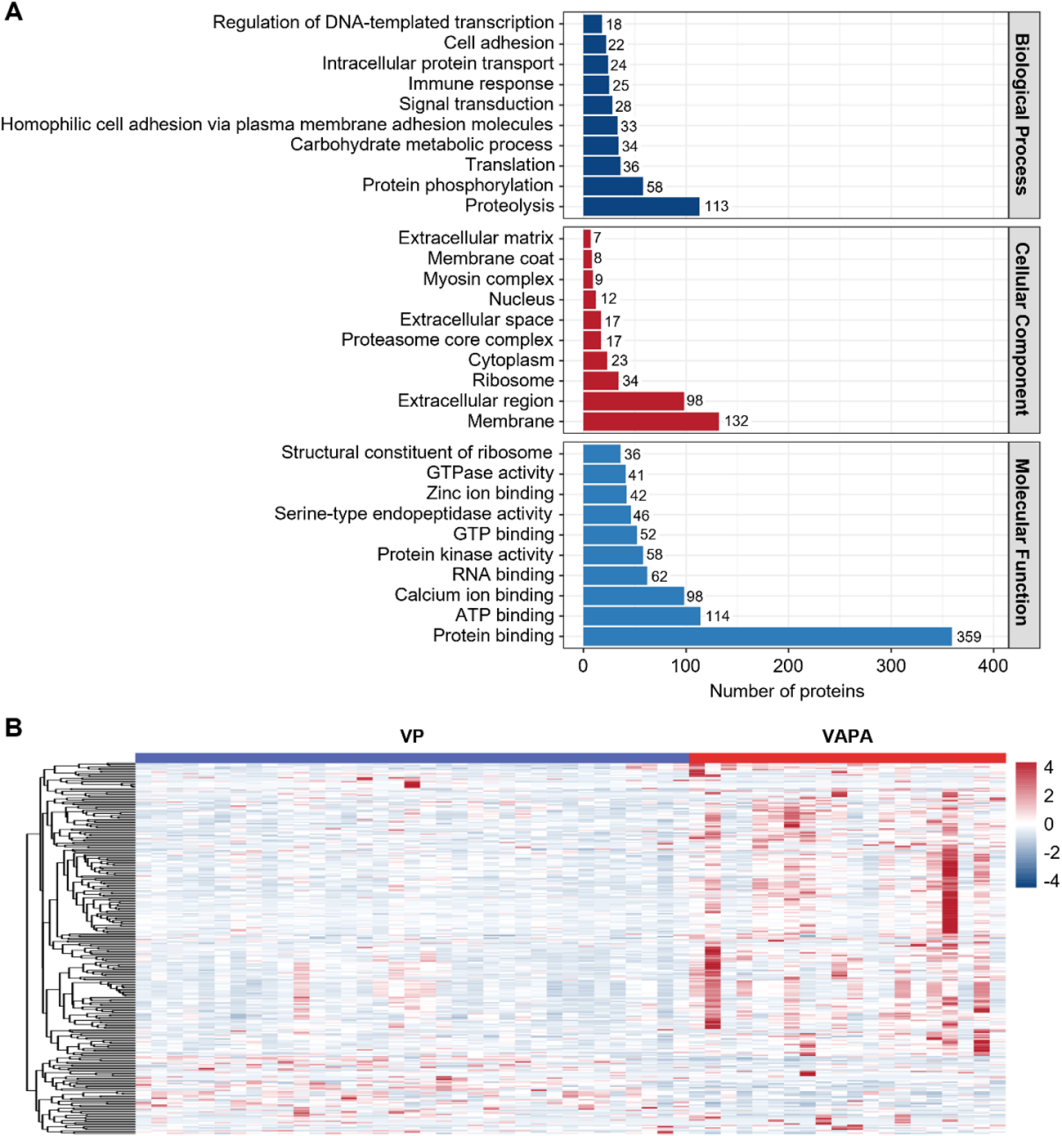
Plasma proteomic profiles of patients with VP and VAPA. **(A)** Bar chart showing functional classification of all identified plasma proteins. **(B)** Hierarchical clustering heatmap of proteins that are differentially expressed between patients with VP and VAPA. Columns represent individual plasma samples, and rows represent differentially expressed proteins. Color intensity from blue to red indicates relative protein abundance ranging from low to high.

To characterize changes in plasma protein expression associated with viral-associated pulmonary aspergillosis, quantitative plasma proteomic profiling was performed using a data-independent acquisition (DIA) strategy in the VP and VAPA groups. A total of 1,930 proteins were identified and quantified. Global Gene Ontology (GO) annotation indicated that these proteins were mainly involved in biological processes such as proteolysis, protein phosphorylation, translation, and signal transduction (**Figure 2A**). Differential expression analysis identified 276 proteins with significantly altered abundances between the VAPA and VP groups. Hierarchical clustering analysis revealed a clear difference of plasma protein expression profiles between the two groups (**Figure 2B**; **Supplementary Table 1**). These differentially expressed proteins spanned multiple functional categories, including immunoglobulins and complement components, chemokines and their receptors, proteases and protease inhibitors, extracellular matrix and adhesion molecules, antioxidant and stress-response enzymes, as well as enzymes involved in carbohydrate, lipid, and amino acid metabolism. These findings indicate broad alterations in immune responses, inflammatory amplification, and tissue remodeling in patients with viral-associated pulmonary aspergillosis.

**Figure 3:**
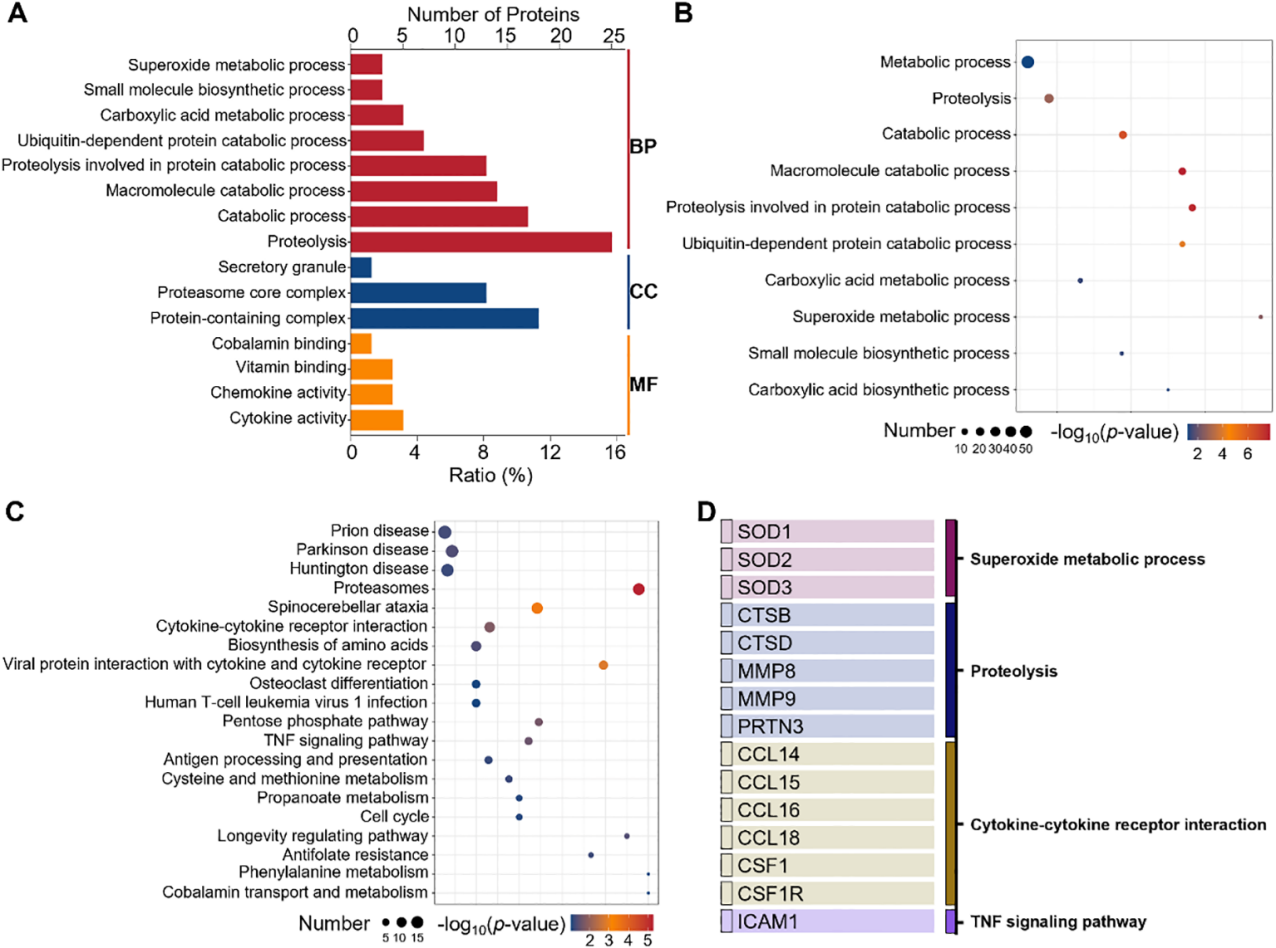
Functional enrichment analysis of differentially expressed proteins between patients with VP and VAPA. **(A)** Bar chart showing Gene Ontology (GO) enrichment results of differential proteins. **(B)** Bubble plot illustrating enrichment of differential proteins in GO biological processes (GO-BP; Top 10). **(C)** Bubble plot showing KEGG pathway enrichment analysis of differential proteins (Top 20). Bubble size represents the number of proteins enriched in each pathway, and color indicates statistical significance (−log_10_ *P* value). **(D)** Network diagram illustrating representative differentially expressed proteins and their associated enriched pathways. SOD1, superoxide dismutase 1; SOD2, superoxide dismutase 2; SOD3, extracellular superoxide dismutase; CTSB, cathepsin B; CTSD, cathepsin D; MMP8, neutrophil collagenase; MMP9, matrix metalloproteinase-9; PRTN3, myeloblastin; CCL14, C-C motif chemokine 14; CCL15, C-C motif chemokine 15; CCL16, C-C motif chemokine 16; CCL18; C-C motif chemokine 18; CSF1, macrophage colony-stimulating factor 1; CSF1R, macrophage colony-stimulating factor 1 receptor; ICAM1, intercellular adhesion molecule 1.

GO enrichment analysis further indicated that the differentially expressed proteins were predominantly associated with biological processes related to protein degradation and oxidative stress regulation, including proteolysis, catabolic process, macromolecule catabolic process, proteolysis involved in protein catabolic process, ubiquitin-dependent protein catabolic process, and superoxide metabolic process (**Figures 3A, B**; **Supplementary Table 2**). These results suggest pronounced activation of protein degradation pathways accompanied by increased oxidative stress burden in patients with VAPA. In parallel, KEGG pathway enrichment analysis showed that the differentially expressed proteins were significantly enriched in several inflammation- and immune-related signaling pathways, including cytokine–cytokine receptor interaction, viral protein interaction with cytokine and cytokine receptor, TNF signaling pathway, and antigen processing and presentation (**Figure 3C**; **Supplementary Table 3**), suggesting heightened immune signaling activity and modulation of antigen presentation in the VAPA group.

**Figure 4:**
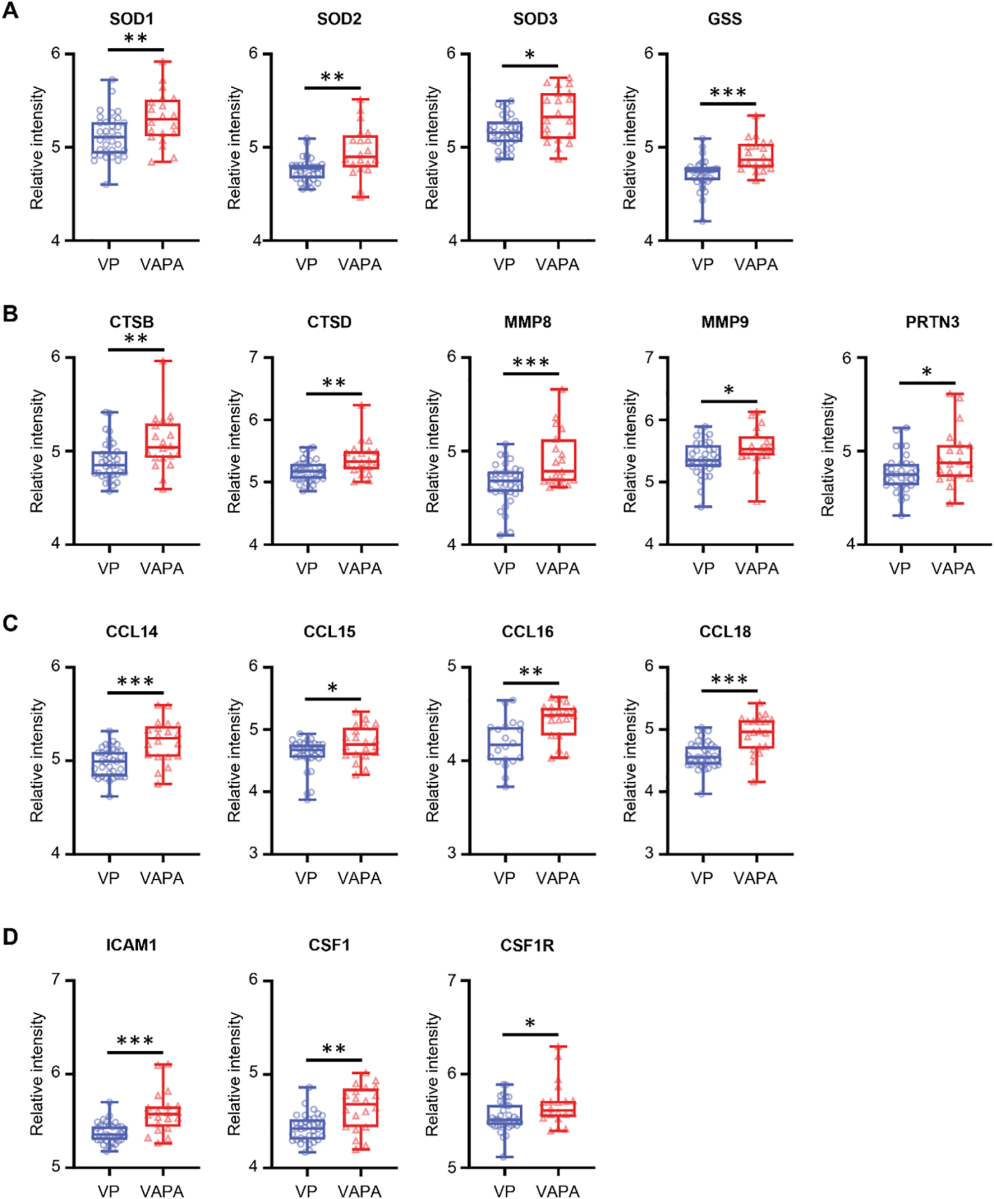
Relative abundance of representative differentially expressed proteins. Relative abundance of representative differentially expressed proteins in patients with VP and VAPA Data are presented as mean ± standard deviation (SD). *p < 0.05, **p < 0.01, ***p < 0.001.

To further characterize proteins contributing to these pathway-level alterations, representative differentially expressed proteins were selected for detailed analysis (**Figure 3D**). Several proteins associated with oxidative stress response, protein degradation, and immune regulation were upregulated in the VAPA group. These included antioxidant defense–related proteins, such as superoxide dismutase [Cu–Zn] (SOD1), superoxide dismutase [Mn] (SOD2), extracellular superoxide dismutase [Cu–Zn] (SOD3), and glutathione synthetase (GSS) (**Figure 4A**). Proteins involved in lysosomal function and proteolysis, including cathepsin B (CTSB) and cathepsin D (CTSD), were also upregulated (**Figure 4B**). In addition, neutrophil-associated proteases, including neutrophil collagenase (MMP8), matrix metalloproteinase-9 (MMP9), and myeloblastin (PRTN3), were upregulated in the VAPA group (**Figure 4B**). Furthermore, multiple proteins involved in immune signaling and cell migration, including C–C motif chemokines CCL14, CCL15, CCL16, and CCL18, were upregulated in patients with VAPA (**Figure 4C**). In addition, intercellular adhesion molecule 1 (ICAM1), macrophage colony-stimulating factor 1 (CSF1), and macrophage colony-stimulating factor 1 receptor (CSF1R) were found to be upregulated in the VAPA group (**Figure 4D**). Overall, the plasma proteomic profile of patients with VAPA was characterized by coordinated changes involving oxidative stress–related proteins, activation of protein degradation systems, and enhancement of immune and inflammatory signaling pathways. These proteomic features were consistent with altered host immune responses and inflammatory states observed in this patient population. To further assess the potential influence of viral etiology, we performed subgroup analyses of representative differentially expressed proteins. In the main VP versus VAPA comparison, proteins related to antioxidant defense, proteolysis, neutrophil-associated proteases, chemokines, adhesion, and monocyte–macrophage regulation were significantly increased in the VAPA group. In the VP versus CAPA comparison, most representative proteins changed in the same direction as in the overall VP versus VAPA analysis. Alth10ough several individual proteins no longer reached statistical significance, GSS, CTSD, PRTN3, CCL14, CCL18, ICAM1, CSF1, and CSF1R remained significantly increased in CAPA patients compared with COVID-19-associated VP patients (**Supplementary Figure 1**). In contrast, most selected proteins did not differ significantly between IAPA and CAPA (**Supplementary Figure 2**). These findings suggest that CAPA retained a subset of the proteomic alterations observed in the overall VAPA group, while IAPA and CAPA showed broadly similar patterns among the representative proteins examined. However, because of the limited sample size, these subgroup findings should be interpreted as exploratory.

### 3.4 Plasma metabolomic characteristics of patients with VAPA

**Figure 5:**
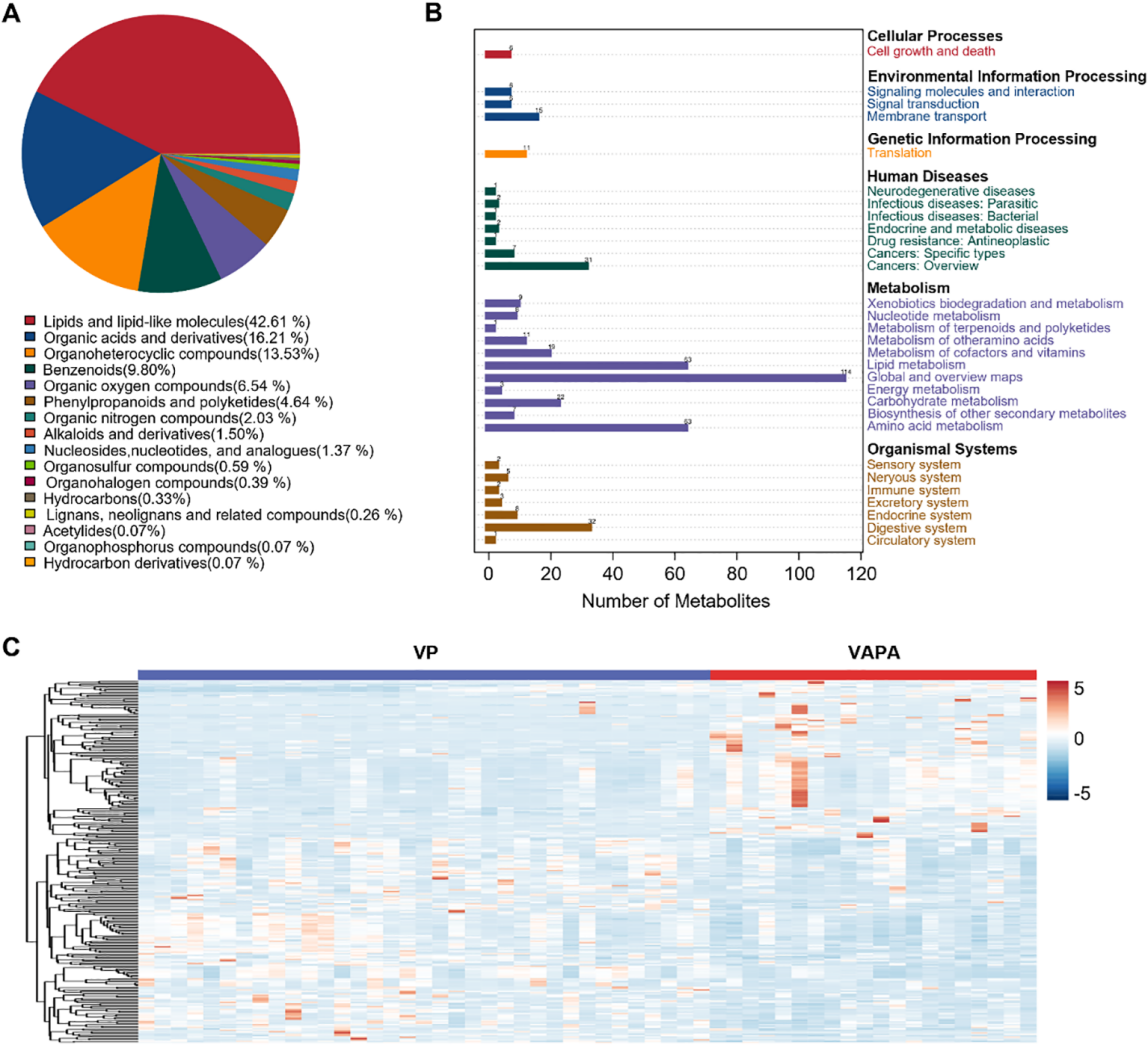
Plasma metabolite composition in patients with VP and VAPA. **(A)** Pie chart illustrating chemical classification of all identified plasma metabolites. **(B)** Bar chart showing KEGG pathway annotations of the identified metabolites. **(C)** Hierarchical clustering heatmap of differentially expressed metabolites between patients with VP and VAPA. Columns represent individual plasma samples, and rows represent differentially expressed metabolites. Color intensity from blue to red indicates relative abundance ranging from low to high.

Based on comprehensive compound library matching using mzCloud, mzVault, and MassList databases, 1,532 metabolites were identified in plasma samples. Lipids and lipid-like molecules represented the largest metabolite class, accounting for 42.61% of all identified metabolites, followed by organic acids and derivatives (16.21%), and organoheterocyclic compounds (13.53%) (**Figure 5A**). KEGG functional annotation indicated that these metabolites were primarily involved in lipid, amino acid, and carbohydrate metabolism, as well as in metabolism of cofactors and vitamins. In addition, a subset of metabolites was annotated to immune system– and endocrine system–related pathways (**Figure 5B**).

**Figure 6:**
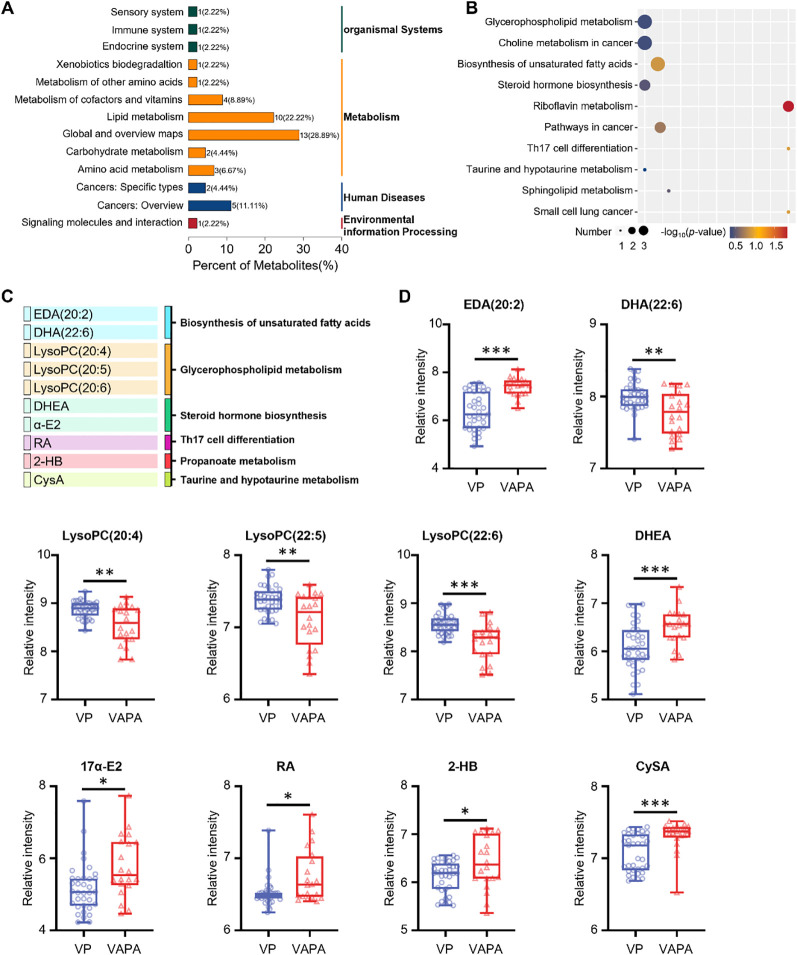
Differential metabolic pathways and representative metabolites between patients with VP and VAPA. **(A)** Bar chart showing KEGG pathway classification of differentially expressed metabolites. **(B)** Bubble plot illustrating KEGG pathway enrichment analysis of metabolites that are differentially expressed between VP and VAPA patients (Top 10 pathways). Bubble size represents the number of metabolites enriched in each pathway, and color indicates statistical significance (−log_10_ *P* value). **(C)** Network diagram depicting representative differentially expressed metabolites and their associated KEGG pathways. **(D)** Relative abundance of representative differentially expressed metabolites in patients with VP and VAPA. Data are presented as mean ± standard deviation (SD). *p < 0.05, **p < 0.01, ***p < 0.001. EDA, cis-11,14-eicosadienoic acid; DHA, cis-4,7,10,13,16,19-docosahexaenoic acid; DHEA, dehydroepiandrosterone; α-E2, estradiol-17α; 2-HB, 2-hydroxybutyric acid; CySA, cysteic acid.

Compared to metabolites in the VP group, 261 metabolites in the VAPA group were differentially expressed and exhibited significantly altered abundance (**Figure 5C**, **Supplementary Table 4**). These differentially expressed metabolites mainly included polyunsaturated fatty acids, lysophospholipids, amino acids and peptide derivatives, steroid compounds, and nucleosides, indicating that patients with VAPA exhibit systemic metabolic alterations involving lipid metabolism, redox regulation, and immune-related metabolic processes. KEGG pathway enrichment analysis further indicated that these differentially expressed metabolites were significantly enriched in glycerophospholipid metabolism, biosynthesis of unsaturated fatty acids, steroid hormone biosynthesis, Th17 cell differentiation, and taurine and hypotaurine metabolism pathways (**Figures 6A, B**, **Supplementary Table 5**). These pathways are associated with lipid metabolic remodeling, redox homeostasis, and immune response regulation. To further characterize the metabolites contributing to alterations in these pathway, representative differential metabolites were selected for detailed analysis (**Figure 6C**). Levels of 2-hydroxybutyric acid (2-HB) and cysteic acid (CySA) were found to be increased in the VAPA group (**Figure 6D**), suggesting enhanced glutathione-related metabolic activity and increased oxidative stress. In addition, metabolites related to lipid and endocrine regulation, including cis-11,14-eicosadienoic acid (EDA), estradiol-17α (17α-E2), and dehydroepiandrosterone (DHEA), were elevated in patients with VAPA, accompanied by increased levels of the bioactive vitamin A metabolite, retinoic acid (RA) (**Figure 6D**). These changes indicate perturbations in fatty acid metabolism, steroid hormone metabolism, and vitamin A–related pathways. Conversely, several long-chain polyunsaturated fatty acids and lysophosphatidylcholines (LysoPCs), including docosahexaenoic acid (DHA, 22:6), LysoPC (20:4), LysoPC (22:5), and LysoPC (22:6), were reduced in the VAPA group (**Figure 6D**), suggesting remodeling of membrane lipid composition and lipid-mediated signaling processes. Collectively, these findings indicate that patients with VAPA exhibit pronounced metabolic reprogramming characterized by dysregulated lipid metabolic pathways, increased oxidative stress, and alterations in endocrine hormone- and vitamin-related metabolism.

To further assess the potential influence of viral etiology on representative metabolomic alterations, we performed additional subgroup analyses. In the main VP versus VAPA comparison, EDA (20:2), DHEA, 17α-E2, RA, 2-HB, and CySA were significantly increased in the VAPA group, whereas DHA (22:6), LysoPC (20:4), LysoPC (22:5), and LysoPC (22:6) were decreased. In the VP versus CAPA comparison, EDA (20:2), DHEA, and CySA remained significantly increased in CAPA patients compared with COVID-19-associated VP patients, whereas DHA (22:6), LysoPC (20:4), LysoPC (22:5), LysoPC (22:6), 17α-E2, RA, and 2-HB no longer reached statistical significance. In the IAPA versus CAPA comparison, most representative metabolites did not differ significantly between the two subgroups, except for LysoPC (22:6) and 2-HB. These findings suggest that CAPA retained part of the metabolomic alterations observed in the overall VAPA group, while IAPA and CAPA showed broadly similar patterns for most selected metabolites, with limited subgroup-related metabolic heterogeneity. These subgroup results should be interpreted as exploratory because of the limited sample size.

**Figure 7:**
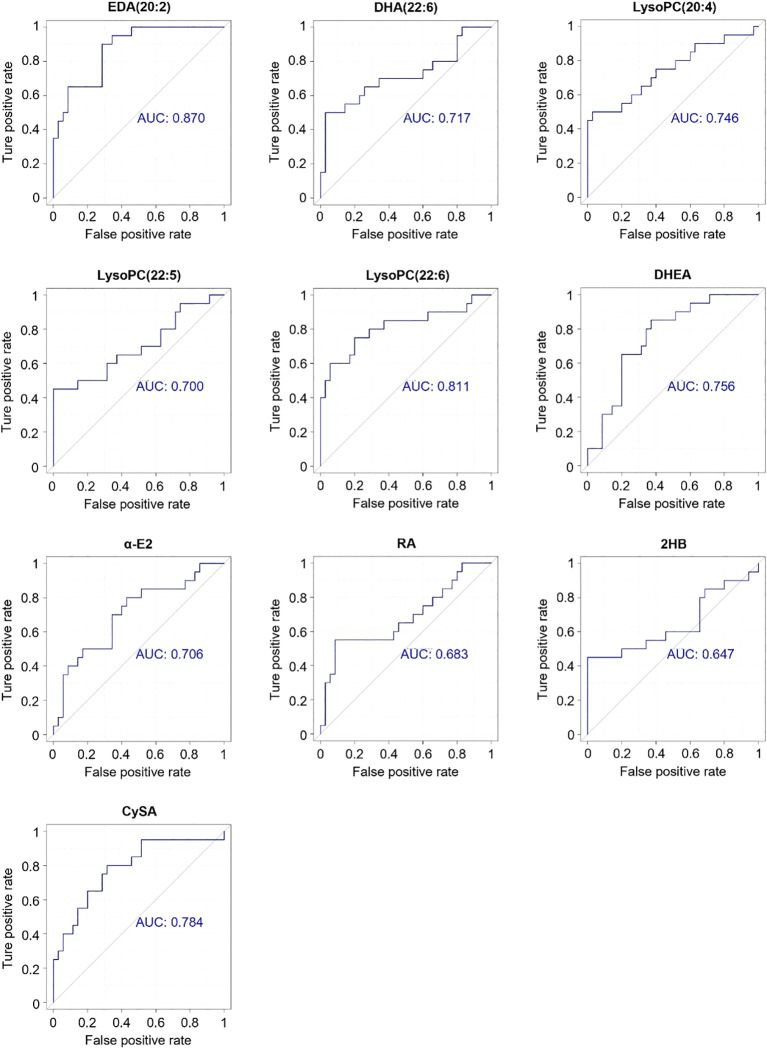
Receiver operating characteristic (ROC) curve analysis of representative differentially expressed metabolites. ROC curves were used to evaluate the discriminatory performance of representative metabolites to distinguish VP from VAPA. Each panel displays the ROC curve and corresponding area under the curve (AUC). An AUC value closer to 1.0 indicates a stronger discriminative ability.

To assess the potential discriminative value of key differentially expressed metabolites that distinguish the VAPA group from the VP group, receiver operating characteristic (ROC) curve analyses were performed for selected representative metabolites (**Figure 7**). Metabolites associated with lipid metabolism, endocrine regulation, and oxidative stress showed varying degrees of discrimination. Among these, EDA (area under the curve [AUC] = 0.870), LysoPC (22:6) (AUC = 0.811), and CySA (AUC = 0.784) exhibited relatively high diagnostic performance, indicating their potential utility as metabolic markers associated with VAPA.

## 4 Discussion

In the present study, we integrated clinical data with plasma proteomic and metabolomic profiling to characterize host responses in patients with VAPA. Compared with patients with VP alone, patients with VAPA exhibited more pronounced systemic inflammatory activation, immune dysregulation, and multi-organ functional impairment at the clinical level. At the molecular level, coordinated proteomic and metabolic alterations, particularly those involving lipid metabolic reprogramming, enhanced oxidative stress, and the activation of immune-related signaling pathways, were observed in patients with VAPA. Taken together, these findings suggest that viral–fungal coinfection is associated with sustained inflammatory activity and disease progression in the context of coupled metabolic and immune dysregulation.

Patients with VAPA showed elevated levels of metabolites related to oxidative stress, particularly those of 2-HB and CySA. 2-HB is an intermediate in the glutathione (GSH) synthesis pathway and its elevated level typically reflects enhanced cysteine availability in response to increased oxidative burden (Sousa et al., 2021). Previous studies reported elevated 2-HB levels in patients with community-acquired pneumonia and COVID-19, wherein they were associated with inflammatory severity and disease progression (Bruzzone et al., 2020; Shi et al., 2021; Zhou et al., 2020). CySA, an oxidation product of cysteine, has been recognized as a metabolic marker of protein oxidation and irreversible oxidative damage (Beltrame et al., 2023). The concurrent elevation of 2-HB and CySA levels in patients with VAPA indicates an intensified oxidative environment associated with fungal coinfection. This metabolic pattern is consistent with activation of GSH-dependent antioxidant responses and aligns with the proteomic findings observed in this study. Multiple antioxidant defense proteins were significantly upregulated, including SOD1, SOD2, and SOD3, as well as the rate-limiting enzyme for GSH synthesis, GSS, in patients with VAPA. The SOD family plays a critical role in redox homeostasis and modulating redox-sensitive inflammatory signaling pathways, including NF-κB–related transcriptional activity (Chen et al., 2023; Zelko et al., 2002). Increased expression of GSS may reflect a compensatory response aimed to enhance availability of GSH under oxidative stress conditions (Atwal et al., 2016; Ristoff and Larsson, 2007; Yang et al., 2019; Zhu et al., 2023). However, under conditions of a high pathogen burden and persistent inflammatory stimulation, such compensatory antioxidant responses may be insufficient to fully mitigate oxidative injury, potentially contributing to sustained inflammatory activity.

Substantial lipid metabolic remodeling is another prominent feature observed in patients with VAPA. Specifically, increased levels of the pro-inflammatory fatty acid, cis-11,14-eicosadienoic acid EDA(20:2), were accompanied by reduced levels of docosahexaenoic acid DHA(22:6) and multiple LysoPCs. EDA(20:2) has been reported to promote the production of pro-inflammatory mediators, including nitric oxide, prostaglandin E_2_, and tumor necrosis factor-α, through arachidonic acid–related pathways, thereby amplifying early inflammatory responses (Huang et al., 2011). In contrast, DHA(22:6) serves as a key precursor for specialized pro-resolving mediators (SPMs), such as resolvins, protectins, and maresins, which are involved in limiting inflammation and supporting tissue repair (Chiang and Serhan, 2020; Ferreira et al., 2022; Lopez-Vicario et al., 2016). The concomitant increase in EDA(22:2) and decrease in DHA(22:6) observed in our study suggests a shift toward sustained proinflammatory lipid signaling. The widespread reduction in LysoPC species further supports this interpretation, as decreased LysoPC levels have been consistently found to be associated with severe infections, sepsis, and unfavorable clinical outcomes (Banoei et al., 2020; Chouchane et al., 2024; Drobnik et al., 2003; Gu et al., 2024). Collectively, these lipidomic changes indicate disruption of fatty acid homeostasis and impaired resolution of inflammation in patients with VAPA.

In addition, elevated levels of 17α-E2, DHEA, and RA were observed in patients with VAPA, suggesting activation of endocrine and vitamin A–related metabolic pathways. Estrogens, including 17α-E2 and estradiol-17β (17β-E2), can suppress NF-κB p65 nuclear translocation and reduce pro-inflammatory cytokine production via estrogen receptor α signaling (Santos et al., 2017). However, elevated estrogen levels have also been associated with increased mortality in patients with sepsis (Feng et al., 2014; Sanchez-Hurtado et al., 2018; Tsang et al., 2016). DHEA modulates cytokine networks and neutrophil function, and its elevation has been linked to poor outcomes in severe pneumonia (Bentley et al., 2019; Mueller et al., 2014). RA, the bioactive metabolite of vitamin A, plays an important role in the regulation of the Treg/Th17 balance and suppression of excessive inflammation via TGF-β/Smad3–Foxp3 signaling (Elias et al., 2008; Mucida et al., 2007; Xiao et al., 2008). RA and its derivatives have also been reported to enhance mucosal antifungal immunity and inhibit the growth of *Aspergillus* (Aggor et al., 2022; Campione et al., 2020, 2021). These metabolic alterations may represent endogenous regulatory responses aimed at maintaining immune homeostasis under viral–fungal coinfection stress, although their protective effects may be insufficient to counteract the overwhelming inflammation observed in severe diseases.

Patients with VAPA exhibited features of immune amplification. Proteins involved in protein degradation and immune processing, including proteasome subunits (PSMA and PSMB), lysosomal proteases (CTSB and CTSD), and neutrophil-derived proteases (MMP8, MMP9, and PRTN3), were significantly upregulated. These changes indicate activation of antigen processing and cellular degradation systems, which may contribute to pathogen clearance but also exacerbate inflammation-induced tissue injury. CTSB has been reported to interact with reactive oxygen species to activate the NLRP3 inflammasome (Zhang et al., 2025), while CTSD has been implicated in macrophage apoptosis and tissue remodeling (Bewley et al., 2011). Both these enzymes are also involved in MHC class II–mediated antigen presentation (Szulc-Dabrowska et al., 2020). Increased levels of MMP8, MMP9, and PRTN3 indicate enhanced neutrophil degranulation, which may compromise tissue integrity despite contributing to antimicrobial defense (Korkmaz et al., 2010; Lerum et al., 2021; Rojas-Quintero et al., 2018; Schuurman et al., 2023). These findings are consistent with elevated neutrophil counts observed in patients with VAPA.

Moreover, several immune regulatory molecules, including ICAM-1, CSF1, CSF1R, and several CCL family chemokines (CCL14, CCL15, CCL16, and CCL18), were upregulated in patients with VAPA. ICAM-1 is a key mediator of leukocyte adhesion and transendothelial migration, and has been reported to increase with disease severity in severe viral infections (Tong et al., 2020; Zhao et al., 2021). The CSF1/CSF1R signaling axis regulates monocyte–macrophage differentiation and survival, and excessive activation can promote M2 macrophage accumulation and fibrotic responses (Lin et al., 2019; Meziani et al., 2018; Munoz-Garcia et al., 2021). Upregulation of multiple CCL chemokines indicates enhanced immune cell recruitment and activation of inflammatory communication networks (Detheux et al., 2000; Hector et al., 2014). Together, these proteomic features suggest that coinfection with *Aspergillus* is associated with reinforcement of chemotactic and immune effector pathways, thereby contributing to sustained inflammatory activity.

At the protein level, a total of 318 proteins were detected only in the VP group, whereas 100 proteins were detected only in the VAPA group (**Supplementary Tables 6**, **7**). However, most of these proteins showed low detection rates. Therefore, these findings were considered exploratory group-specific detection features rather than robust group-specific biomarkers. At the metabolite level, no metabolites were exclusively detected in either the VP or VAPA group. An important issue is whether the molecular alterations observed between VP and VAPA mainly reflect fungal co-infection or are influenced by the underlying viral etiology. In this cohort, all VP patients had COVID-19-associated viral pneumonia, whereas the VAPA group included both IAPA and CAPA cases. Additional subgroup analyses showed that several representative proteins, including GSS, CTSD, PRTN3, CCL14, CCL18, ICAM1, CSF1, and CSF1R, as well as metabolites including EDA (20:2), DHEA, and CySA, remained significantly altered in CAPA patients compared with COVID-19-associated VP patients. These findings suggest that part of the VAPA-associated proteomic and metabolomic changes may be related to pulmonary aspergillosis superimposed on severe viral pneumonia rather than being explained solely by viral etiology. However, comparison between IAPA and CAPA showed that most representative proteins and metabolites did not differ significantly between the two subgroups, except for LysoPC (22:6) and 2-HB, suggesting shared downstream host-response features with limited subgroup-related metabolic heterogeneity. Larger cohorts stratified by viral etiology are needed to define shared and virus-specific molecular signatures in VAPA.

Several limitations of this study should be acknowledged. This was a single-center retrospective study with a relatively small sample size, particularly in the VAPA group, which may limit the statistical power and restrict subgroup analyses. Although exploratory subgroup analyses were performed, the small number of IAPA and CAPA patients limited the ability to distinguish shared VAPA-associated molecular changes from subtype-specific or virus-related alterations. Therefore, these subgroup findings should be interpreted cautiously and validated in larger and more balanced cohorts. The cross-sectional design did not allow for assessment of temporal or causal relationships between molecular alterations and disease progression. In addition, plasma samples may not fully reflect local metabolic and immune processes within the pulmonary microenvironment. Finally, functional validation experiments were not performed, and the mechanistic roles of key metabolites and proteins require further investigation using experimental models. Therefore, these findings warrant validation in larger multicenter cohorts and mechanistic studies.

## 5 Conclusion

In summary, this study systematically characterizes the host metabolic and immune features associated with viral-associated pulmonary aspergillosis. These findings provide insights at the molecular level into host responses associated with viral–fungal coinfection and may assist in future studies aimed at early risk stratification, identification of metabolism–immune–related biomarkers, and exploration of targeted therapeutic strategies.

## Data Availability

The datasets presented in this study can be found in online repositories. The names of the repository/repositories and accession number(s) can be found in the article/**Supplementary Material**.
