# Supplementary material for: Amplified inflammatory and immune responses in viral-associated pulmonary aspergillosis
Source: Front Cell Infect Microbiol. 2026 May 28;16:1850127. doi: 10.3389/fcimb.2026.1850127 (PMC13253414; doi:10.3389/fcimb.2026.1850127)
Supplement: Supplementary file 1 [file Table1.docx]

**
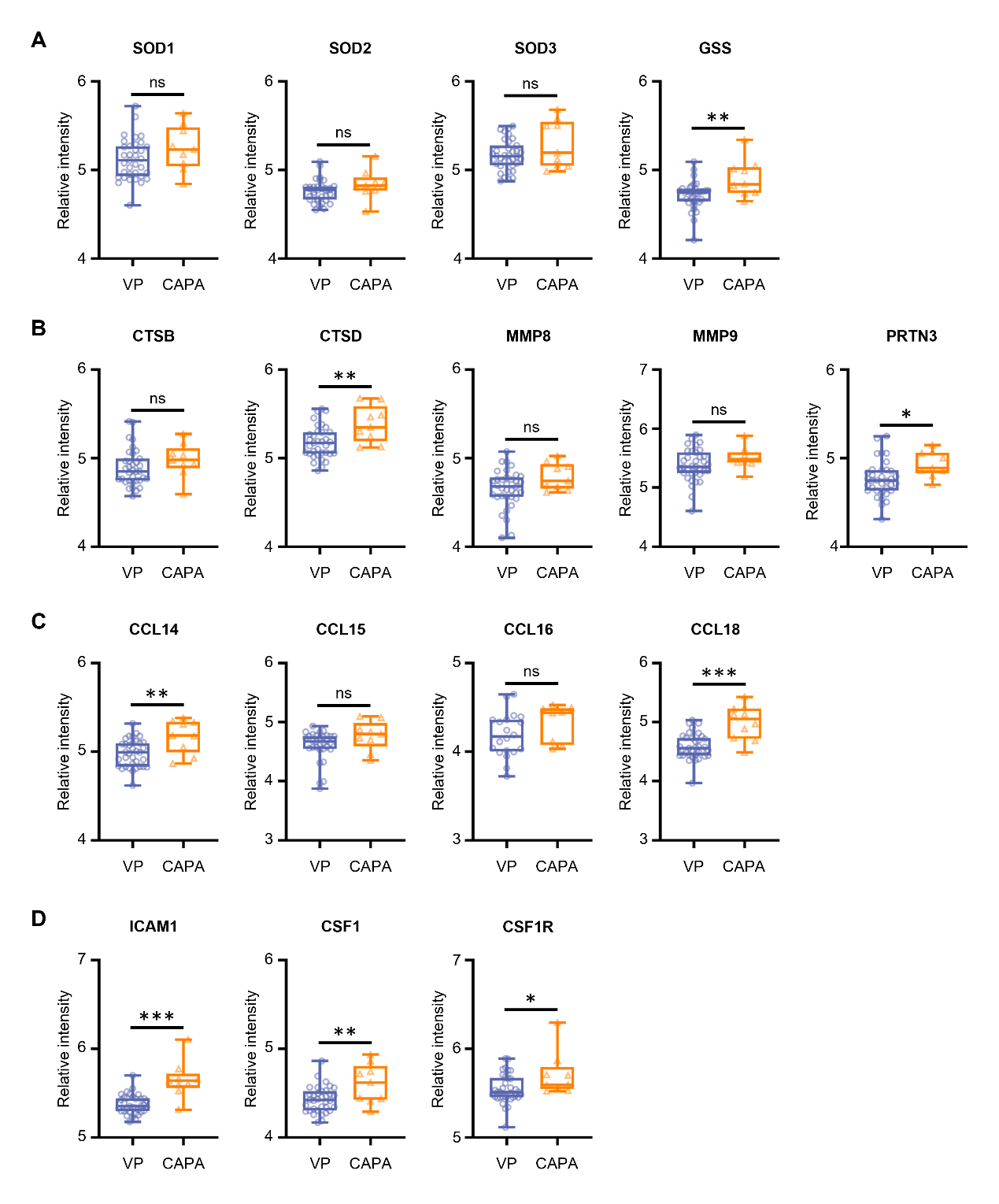
**

**Figure S1. Relative abundance of representative differentially expressed proteins.**

Relative abundance of representative differentially expressed proteins in patients with VP and CAPA Data are presented as mean ± standard deviation (SD). **p* < 0.05, ***p* < 0.01, ****p* < 0.001.
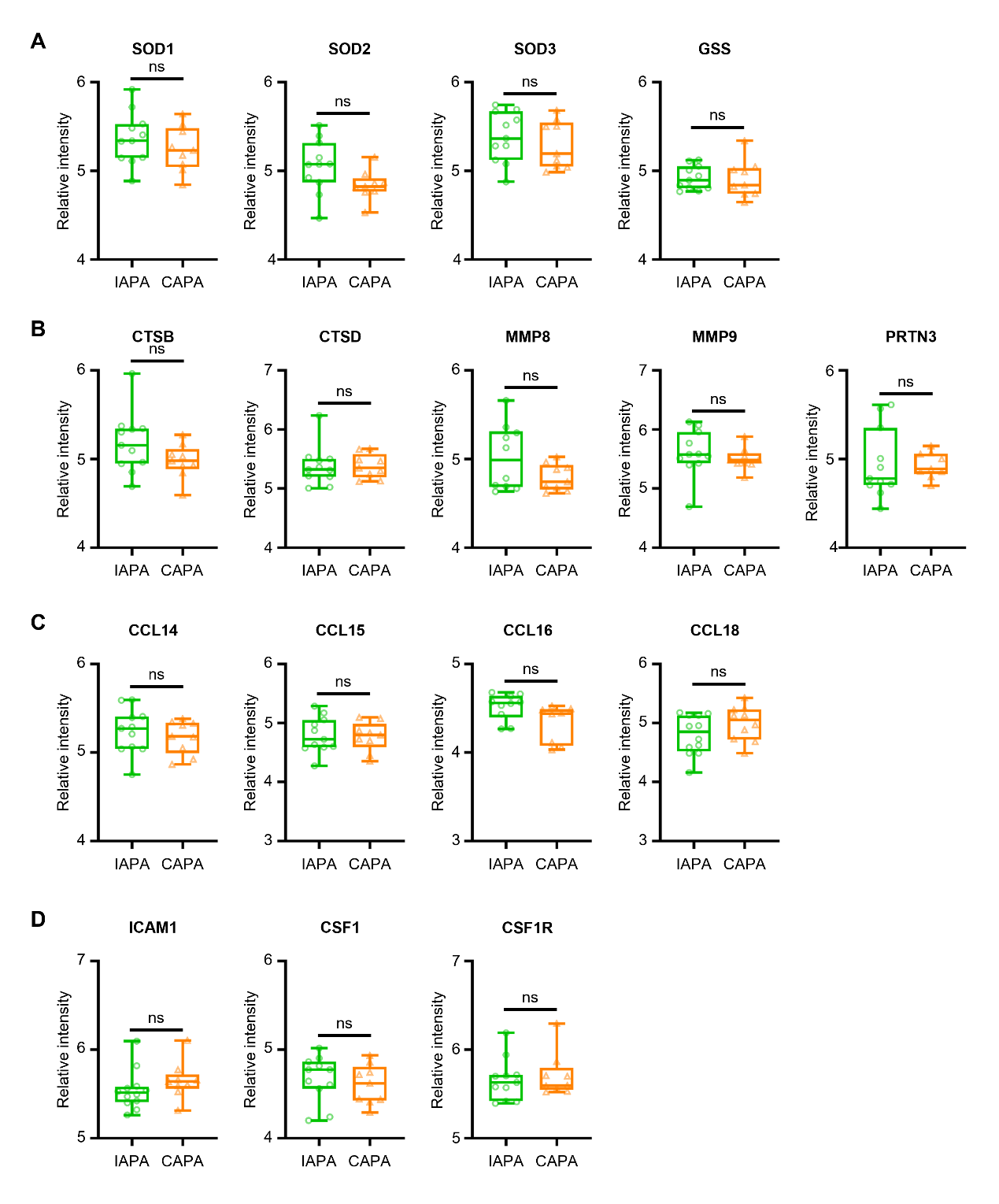


**Figure S2. Relative abundance of representative differentially expressed proteins.**

Relative abundance of representative differentially expressed proteins in patients with IAPA and CAPA Data are presented as mean ± standard deviation (SD).


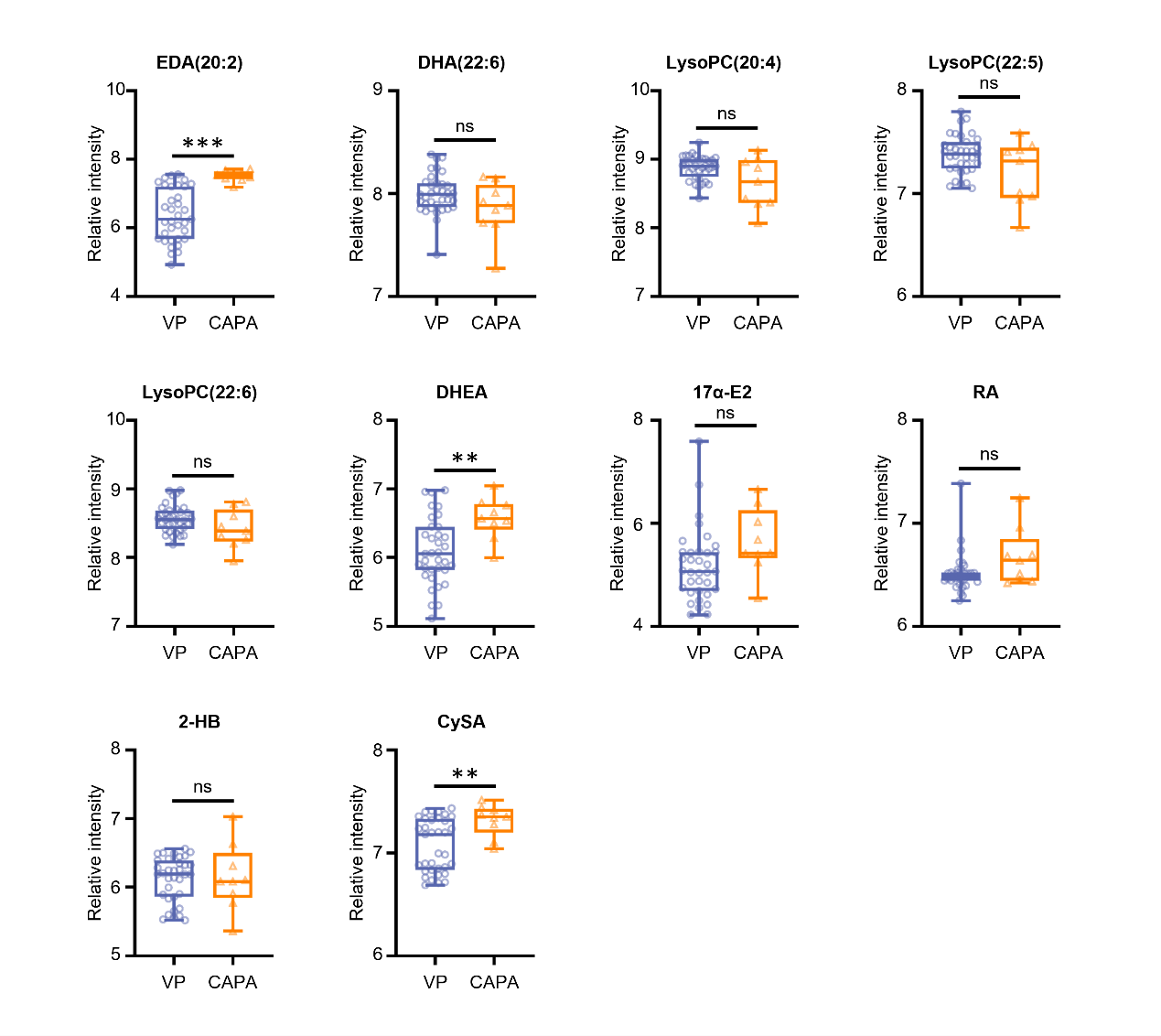


**Figure S3. Relative abundance of representative differentially expressed metabolites.**

Relative abundance of representative differentially expressed metabolites in patients with VP and CAPA. Data are presented as mean ± standard deviation (SD). ***p* < 0.01, ****p* < 0.001.
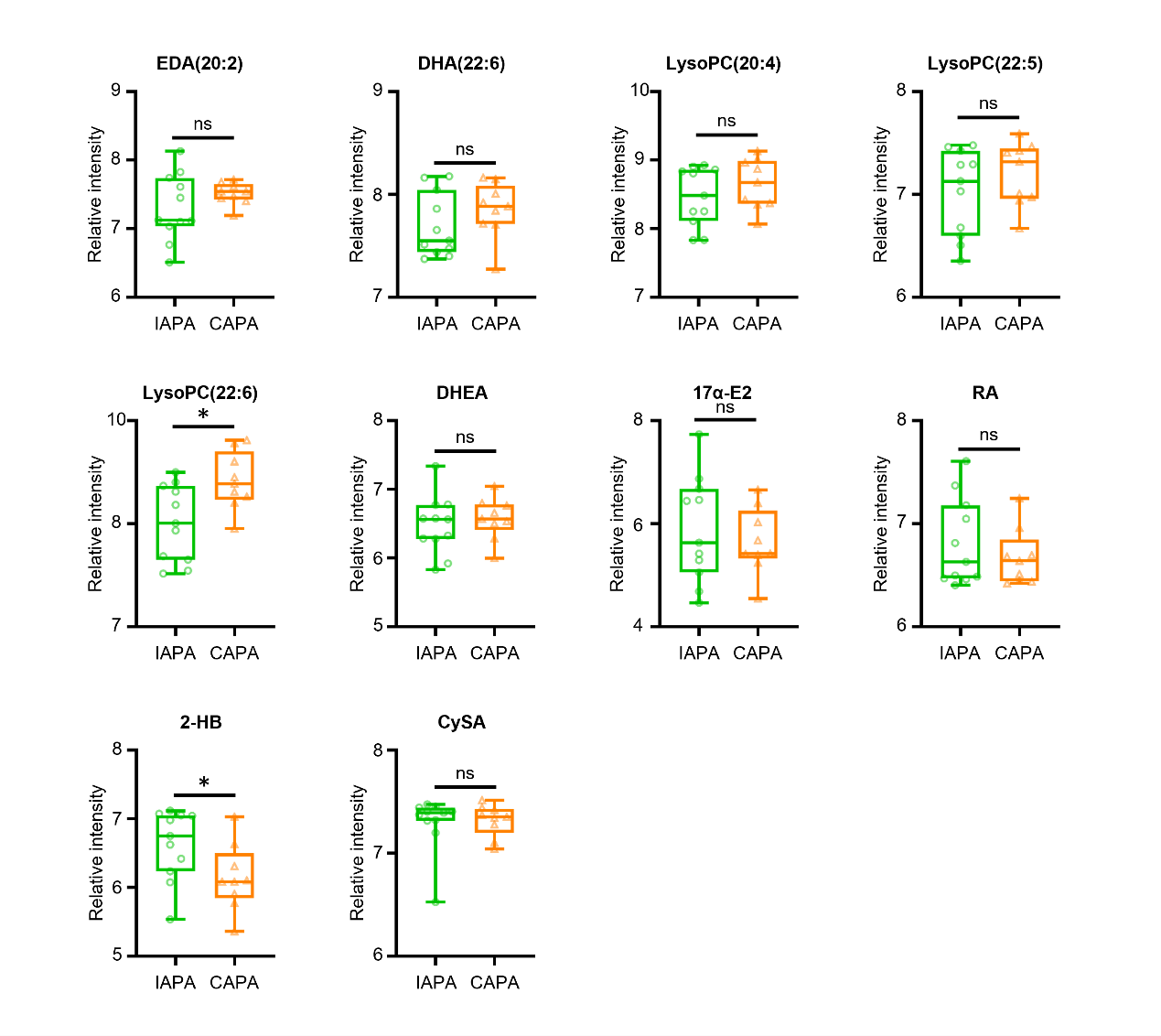


**Figure S4. Relative abundance of representative differentially expressed metabolites.**

Relative abundance of representative differentially expressed metabolites in patients with IAPA and CAPA. Data are presented as mean ± standard deviation (SD). **p* < 0.05.
